# Ontology construction and application in practice case study of health tourism in Thailand

**DOI:** 10.1186/s40064-016-3747-3

**Published:** 2016-12-20

**Authors:** Chantana Chantrapornchai, Chidchanok Choksuchat

**Affiliations:** 1 Department of Computer Engineering, Faculty of Engineering, Kasetsart University, Bangkok, 10900 Thailand; 2 Department of Computing, Faculty of Science, Silpakorn University, Nakhon Pathom, 73000 Thailand

**Keywords:** Ontology engineering, Health tourism, Hua-Hin tourism, Knowledge management

## Abstract

Ontology is one of the key components in semantic webs. It contains the core knowledge for an effective search. However, building ontology requires the carefully-collected knowledge which is very domain-sensitive. In this work, we present the practice of ontology construction for a case study of health tourism in Thailand. The whole process follows the METHONTOLOGY approach, which consists of phases: information gathering, corpus study, ontology engineering, evaluation, publishing, and the application construction. Different sources of data such as structure web documents like HTML and other documents are acquired in the information gathering process. The tourism corpora from various tourism texts and standards are explored. The ontology is evaluated in two aspects: automatic reasoning using Pellet, and RacerPro, and the questionnaires, used to evaluate by experts of the domains: tourism domain experts and ontology experts. The ontology usability is demonstrated via the semantic web application and via example axioms. The developed ontology is actually the first health tourism ontology in Thailand with the published application.

## Introduction

Ontology is an important element underlying the semantic web technology. It contains the domain-specific knowledge to increase the powerfulness of a particular search engine. The ontology design is very important since it affects the search effectiveness and efficiency. With the good property characteristic design, the inference engine can infer new knowledge from existing ones, returning more related results.

Designing ontology and evaluating it are challenging tasks. Ontology contains several concepts in the object-oriented style. The relationships are the properties which map from a domain to a range and can be viewed as functions or relations. The property characteristics are needed to increase the possibility of the knowledge inference.

Designing particular ontology requires domain knowledge. Moreover, the designer must know about the object concept, the hierarchy of subclasses, the property mapping, and property characteristics. These are required for the inference engine. After designing and inputting instances or individuals, it is questionable whether the design is complete according to the specification.

Tourism is one of the interesting application domains since tourism industry can attract tourists to a country or region which can increase the local or domestic income. With a good information system and Internet infrastructure, the search can facilitate tourists to find the right travel information and accommodation.

Semantic web has been applied in many applications including tourism. It has been used in Morocco, Hong Kong, China, Germany, and etc. Though there are many existing tourism ontologies, each of them has different focuses. In this research, we are interested in developing a semantic web for health tourism in Thailand. The prototyped ontology is based on the health tourism in Hua Hin district. We discuss the design process and experiences focusing on the health tourism ontology engineering, starting from information gathering, ontology conceptualization and evaluation, until the application deployment.

Our development has two unique characteristics.

1. Current ontology around is about a general tourism concept which focuses on attractions, hotels, and etc. The ontology example on health tourism, to our knowledge, is not available.
2. This work is a pioneer work in gathering extensive Hua Hin health tourism information, and classifying it using ontology. We focus on experiences in gathering such information in practice and building the ontology as well as the prototype application.

The next section presents a brief background on ontology, Resource Description Framework (RDF), and Web Ontology Language (OWL). Also the definition of the health tourism domain is presented as well as related studies. Third section presents the overall methodology. Fourth section presents the ontology design and the evaluation process. Fifth section demonstrates the sample application and discussion in sixth section. Conclusion is presented in final section.

## Background

This section presents some background related to the research. It includes backgrounds in ontology, RDF, and OWL. Next, we present the definition of health tourism, and its context of Thailand. At last, we discuss the literature area in the field.

### Ontology, RDF, OWL

Tim Berners-Lee presented the future web concepts and published them in Scientific American 2001 (Berners-Lee et al. 2001) known as “Semantic Web”. The purpose of this concept is to enable machines to comprehend semantic documents and data that are enriched by the convention. Three components are identified: ontologies, knowledge representation and agent as essential to function. The semantic web development has been done in many domains such as tourism, languages, organization.

The core standard is RDF for knowledge representation (Auer et al. 2007). RDF is a data model consisting of a triple (i.e. subject, predicate, object), containing information about web resources. OWL used along with RDF is an ontology language for a semantic web with formally-defined meaning.

Since OWL became a W3C standard (Bechhofer et al. 2004), there has been a notable increment in the number of ontologies. In OWL1 species, there are three official sublanguages: OWL Full, OWL DL and OWL Lite. The study of OWL DL has become an important aspect of ontology representation for any inference problem in the semantic web. The OWL DL is essentially the description logic (DL), SHOIN^(D)^. It offers a high level of expressivity. For example, it provides full negation, disjunction, and a restricted form of universal and existential quantification of variables (Motik et al. 2005; Beek and Horrocks 2005).

OWL DL language constructs can be represented using classes, roles, individuals, class membership and role instances, owl:Thing and owl:Nothing, class equivalence, class conclusion, negation of classes, disjointness, conjunction, disjunction, role restriction using *owl:allValuesFrom* and *owl:someValuesFrom*, *rdfs:domain* and *rdfs:range*.

### Health tourism

According to the World Health Organization (WHO), health is a state of complete physical, mental and social well-being and not merely absence of disease. Health tourism is the used of medical services away from home or the travel for the purpose of healing, obtaining medical services of health improvement, and etc.

Health tourists are those who travel for the above purposes. In Thailand, health tourism is divided in two kinds based on purposes.

1. Health healing: it is the travel whose purpose is to restore health, and cure diseases, including cosmetic surgery, and dental services. This is called *medical tourism*.
2. Health promotion: it is the travel whose purpose is to increase health strength in the tourist attraction area. Examples are spa, aroma therapy, Thai herb sauna or stream, and body massage. Supplementary services may be body detox, meditation, yoga, and nutrition consulting. This is also called *wellness tourism*. In some texts, it is divided into *spa* and *wellness tourism.*

The spa business is a major business in Thailand’s wellness tourism which is included in the term “health business” according to the Act of legislation of Public Health Ministry (B.E. 2509).

The business provides a place for health or beauty maintenance. The health business is categorized as:

1. Spa business for health. It is a type of business that uses water and massage for treatment. There can also be facilities such as nutrition advice, herb sauna and stream, meditation, yoga and alternative medicines.
2. Massage business for health. The business focuses on massaging for health, and for relaxing according to massage science. It does not provide a bathing area.
3. Massage business for beauty. Examples of this type of business are hair salons or beauty salons. It contains different massage type according to the massage science, which can increase personal beauty. However, it does not provide a bathing area.

Hua Hin is one of the popular districts in Thailand that is close to Bangkok. It comprises many interesting attractions especially beautiful beaches. There are many local and international tourists. There are many famous resorts and spas as well as destination spas. It is expected to be the next health tourism area in addition to Bangkok.

In Hua Hin, there are lots of medical tourism and wellness or spa tourism businesses. For medical tourism, there are also many famous hospitals and clinics, dental clinics, beauty clinics, pharmacy, and etc. It is one of the destinations for wellness spas as promoted by the Thai Government with the policy “Medical Hub of Asia” (Thai Board of Investment 2012). In Hua Hin, there are four kinds of wellness spas:

1. *Hotel and resort spa* It is a kind of the spa situated in a hotel. The main business is the hotel and resort while the spa is a facility of the hotel.
2. *Destination spa* It is the spa which provides packages for tourists who intend to take a spa course. The tourists must attend a course which may require 2 or more night stays at the resort. A very famous one in Thailand is Chiva-Som (http://www.chivasom.com) which is located in Hua Hin.
3. *Day spa* It is a kind of spa which provides various services. The treatment or service can be applied and there is no need for an overnight stay. On the contrary, the hotel and resort spa can be a day spa since an outside hotel guest can take a visit.
4. *Medical spa* It is the facility that lies between the medical clinic and the day spa. It must be operated under a qualified medical team in many related fields including cosmetic surgery, nutrition, and etc.

### Related work

First, we discuss the work in information or knowledge engineering in tourism. Secondly, the methods to evaluate the ontology are studied, and then the applications of tourism that use the ontology are discussed.

#### Ontology extraction in tourism

A lot of work studied ontology extraction approaches. They differed in the application domain, the techniques, the target documents, automatic or semi-automatic approach.

Meersman et al. (2009) presented a way to write the ontology documents (Meersman et al. 2009; Ruiz-Martínez et al. 2011). They focused on how ontology requirement was gathered and the ontology specification was written formally. The key activities were the search and reuse of existing knowledge resources and ontological resources. Also, the verification and validation were considered.

Karoui et al. (2004) proposed the automatic method for the ontology discovery for the tourism application. They proposed the ontology building process from HTML documents which is a complement to the Aussenac-Gilles approach. It used similarity and clustering techniques to group words to define the hierarchy. The processes were corpus preparation, Aussenac-Gilles methodology for ontology building, discovery approach based on clustering considering the HTML structure.

Ogata (2001) described a framework to construct the formal ontology based on web documents. The method was based on logics, and web technology, XML, and NLP. Mouhim et al. (2011) presented the knowledge management approach based on ontology. They used Morocco tourism ontology. The approach considered Mondeca tourism ontology, OnTour ontology (Siorpaes et al. 2004), etc. Then, the vocabulary was constructed from thesaurus by the United Nation World Tourism Organisation (UNWTO). The category was established and social platforms were examined. Next, the ontology was built using the tool and verified.

Tang and Cai (2010) presented the domain ontology construction from unstructured texts. The approach started from pre-processing of the text to extract keywords or preserved terms and composed simple and compound statements. The descriptive logic (DL) was used to represent the knowledge. At last, the ontology was generated.

Sigala et al. (2007) presented an approach for the creation of the e-tourism domain (Sigala et al. 2007). The process contained four steps: NLP and corpus processing, named entity recognition, ontology population, and consistency checking stages. The first stage used POS Tagger, and syntactic parser, while the second stage used Gazetter and Transducer. The last stage used OWL2 reasoner.

Alani et al. (2003) proposed an automatic extraction of knowledge from the web documents. The domain of impressionist artists and their painting was selected as a prototype for the process. The knowledge extraction was done based on the HTML structure, pattern rule extraction, or machine learning. The corpora such as WordNet and lexical database, and GATE were used to extract name entities and relationships.

Daramola, Adigun, and Avo built ontology for a tourism recommendation application (Daramola et al. 2009). The developed ontologies were Destination Context Ontology and Accommodation Ontology. The usability evaluation was collected from 15 users of the recommendation service.

Gouveia and Cardoso (2007) presented an integration of the tourism information in ontology. The architecture consisted of five layers: semantic layer, mapping layer, syntactic layer, and external data sources. The tourism ontology was at the semantic layer.

Our ontology is different from all the ontologies above. We combine a spa tourism concept and a general tourism concept. Moreover, our concept drills down to the detail of each activity of spa tourism that is appropriate in Thailand.

#### Ontology evaluation

Supekar (2005) presented the peer-review approach for reviewing the ontology. The approach provided the qualitative ratings of the ontology content. It is the qualitative research that evaluated the content of an organizational ontology, developed within a large Brazilian energy utility company. The evaluation process consisted of a set of questionnaires, based on a multi-disciplinary approach, of a prototype system. The methodology contained four phases: determining the research tools, collecting data for the design of the ontology, building the ontology, and evaluating the ontology.

Almeida (2009) proposed a way to evaluate ontology using questionnaires. The questionnaires contained three kinds of questions: competency questions, information quality, and educational objectives.

Mugellini et al. (2011) presented the quality assurance framework for ontology construction. The approach was based on Hozo reasoner for consistency verification. The prototype ontology was a sustainability science and clinical ontology. For the content, they evaluated the relationships among concepts using concept maps.

Tankeleviciene and Damasevicius (2009) described the characteristics of domain ontology. They proposed two methods for evaluation: (1) a method for an expert-based evaluation of the ontology content, (2) a model and a collection of technical metrics to evaluate using the structural complexity of ontology. Their application was web-based learning. The complexity was analyzed using 7DO models. The expert evaluation was based on completeness, consistency, conciseness, preciseness and clarity.

Lehmann et al. (2011) presented the evaluation of class expression. They created OntoWiki plugin for DL-Learner functionality and tested ontologies using DL expressivity.

Kehagias et al. (2008) presented a method for evaluating the ontology. The conditions used to check the completeness had the following criteria: concept and property hierarchy, module subtraction, documentation and visualization, definition of ranges for property values, disjointness restrictions and adherence to naming conventions.

Five basic internal layers were considered for evaluation: lexical/vocabulary layer, structural/architectural layer, representational/semantic layer, data/application layer, and philosophical layer. Basic external dimensions were (1) user dependence: how many users depend on the ontology? (i.e., what is the impact of changes to the ontology? should this be avoided or is it simple to implement?) (2) is the ontology used as a medium of information exchange across distinct communities? (3) is it documented? If so, in which form? (natural language, UML, logical spec, and etc.) (4) is it a national or international standard? (5) Is it a de facto working standard for some community? (6) usability layer. The approach was applied to ASK-IT (TourismAndLeisureOntology).

According to the above study, we apply two kinds of evaluation: the evaluation by automated reasoning and the evaluation by the experts. For the first type, Pellet and RacerPro reasoners which are plug-into Protégé ontology editor are used. The evaluation focuses on inferring the concept from the created ontology assertion. For the second type, the proposed ontology is evaluated by experts’ questionnaires where the focus of the questions is completeness and documentation.

#### Related work in semantic web and tourism semantic web

Currently, several tourism projects are based on ontology. Harmonized project (aka Harmonize project) (Dell’Erba et al. 2002, 2005; Foder and Werther 2005) is one of the semantic platform which provides a shared ontology and facilitates the semantic cooperation between the tourism business sectors in European countries.

SATINE (Dogac et al. 2004) is a the famous framework which extends Global Distribution System (GDS) connecting Online Travel Agent (OTA) between semantic webs for distributed web service platforms. Each tourism service needs the registry to be included for the automatic search.

Bottari (Balduini et al. 2012) combined the social network such as Twitter to the ontology which can recommend about the rated restaurants for a given time period. Jakkilinki, Sigala et al. (2007) developed an application for a tour planner with an intelligent approach using the designed ontology. Cardoso (2006) proposed to generate the semantic web process dynamically. It uses web services to gather information. Many services were composed. E-tourism is used as an application for the approach.

EIFFEL (2006) by Mondeca is a tourism search engine developed by French National Software Technology (Mondeca 2006). The goal is to promote regional tourism. It uses semantic-oriented widgets to construct a semantic web portal.

Developing health tourism ontology may be related to many existing ones. For example, in Table 2 is a sample list of ontology we have surveyed. Most of them are general tourism concepts with different focuses. Column “DL expressivity” shows the reasoning of each ontology.

Consider existing health tourism web sites in Thailand, such as: http://www.mymedholiday.com/country/thailand/hua-hin, http://www.thailandmedtourism.com/DestinationDetail/99/2579/Hua-Hin, http://www.thaimedtour.com/content/163/Hua-Hin-Hospital-/, http://www.health-tourism.com/, etc.

The first three are local sites while the last one is not. The first two sites list famous hotels with branded spas, well-known hospitals and clinics. They have a good information presentation but the variety of data is limited. The third one presents only information about hospitals and clinics. It contains hospitals and clinics all over the world. Only paid registered businesses are presented. Thus, our work is the pioneer work to gather health tourism information and build the ontology for Thailand. We aim to publish these data as open linked data in the future.

## Methods

The two popular approaches in building ontology are *METHONTOLOGY* (Fernández-López et al. 1999; Gómez-Pérez 1996), and NeOn (Suárez-Figueroa et al. 2008). The methodology of *METHONTOLOGY* is more appropriate to us since it focuses on a method to build ontology from scratch, and partly reuses other ontologies while NeOn presents nine scenarios for building the ontology network where most of them consider the scenarios for ontology reuse.

**Fig. 1:**
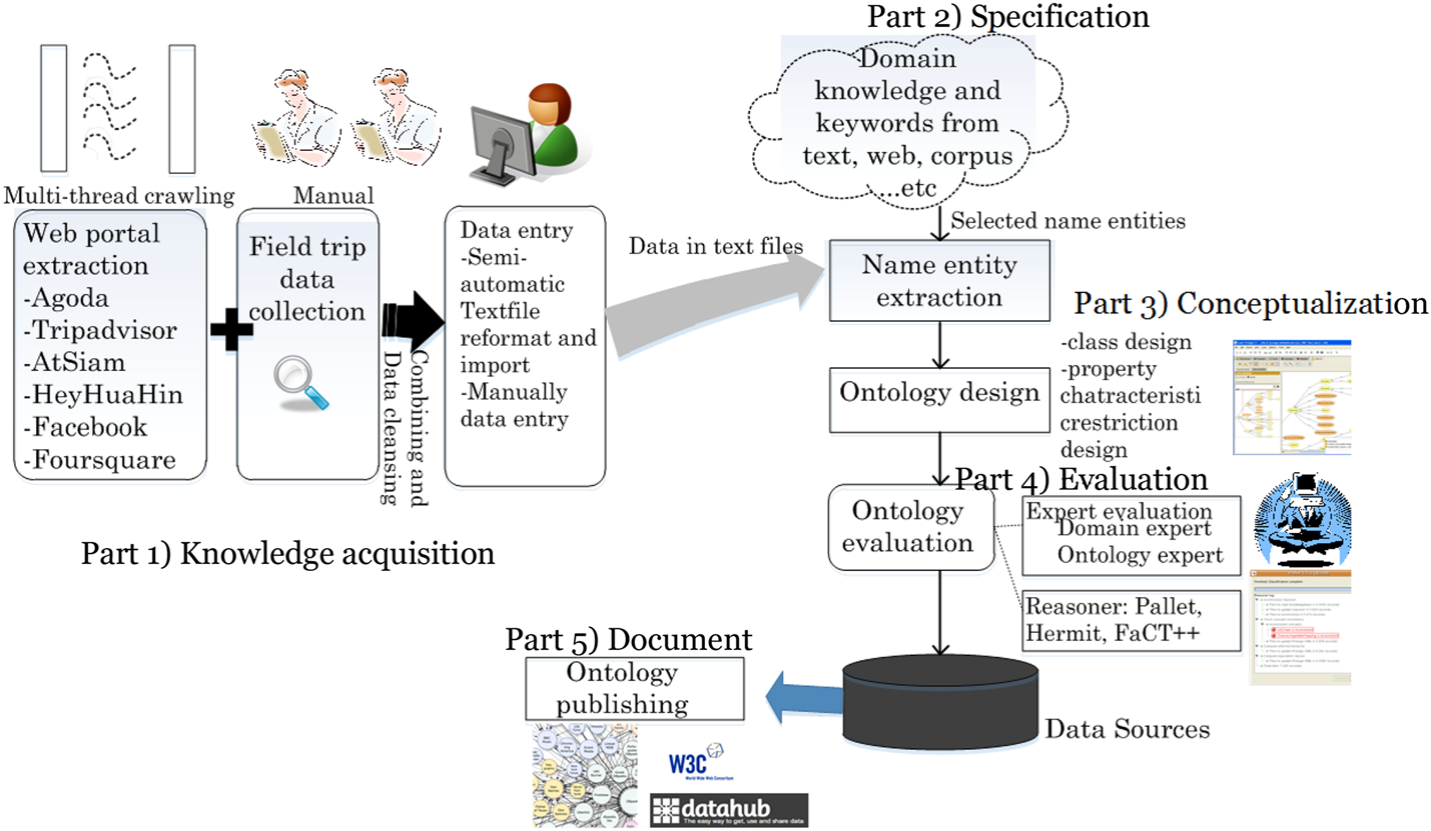
Overview process of ontology building

Recall that METHONTOLOGY consists of five steps: specification, knowledge acquisition, conceptualization, integration, and implementation. In Fig. 1, these phases are located. For part (1) in the figure, knowledge acquisition, two kinds of information gathering are used: the semi-automatic approach and the manual approach. The semi-automatic approach is to explore the existing tourism data on the Internet. Typical websites for Hua-Hin tourism are gathered and the information provided by each site is studied. We build a parallel program to extract information from the websites. The extracted information is the spa information provided by hotels and resorts. For the specific details of the day spas, the medical places, the destination spa, and the spa packages, we need a field trip to collect the information and some personnel to search particularly at their official websites. Also, the information about the spa business registered at the Ministry of Public Health is requested. These are the information in hardcopies and in Excel files.

Part (2) is relevant to specification, where a corpus of health tourism definition and the related terms, categories including natural products, such as Mueller and Kaufmann (2001), Caballero-Danell and Mugomba (2007), ISPA (Cunningham et al. 2014; Põld; Constantinides 2011; Smith and Puczko 2009) etc. are studied. Several existing tourism ontology or related ontology like Dell’Erba et al. (2002, 2005), Foder and Werther (2005), Knublauch, Siorpaes et al. (2004), Sigala et al. (2007), Ou (2008) are studied. The goal is to extract name entities to derive classes, subclasses, and individuals for the ontology design. The classification about health tourism business is also reviewed. In part (3), we start to extract major attributes or keywords from the previous collected data for conceptualization of ontology. Some grouping is created and synonyms are gathered. The property may be grouped into subclasses such as the product of spa, and spa area.

Lastly, in part (4), the ontology is evaluated by both experts and reasoners. After that, in part (5), the documents of ontology are prepared for publishing.

## Ontology conceptualization

Previous tourism ontology in the literature has different focuses. Some focuses on accommodation while others focus on cultural objects, packages, and etc. Also, the tourism in each country has its own unique characteristics.

In particular, there are some special tourism attractions and lifestyle (Mili et al. 2011) for some regional area. There are several kinds of tourism attractions in Thailand. The cultural styles embed traditional Thai lifestyles in the past. For example, the housing and decoration styles are preserved as the traditional Thai housing style. This is shown as the architecture style of the building. Thai massage is also a unique massage type. It includes compress massage, reflexology massage, Ayurveda massage, and Chaleoysak massage.

From the information gathering approach described in “Appendix 1”, we start from designing classes and hierarchy which are subclasses (Gouveia and Cardoso 2007). Also, relations must be described, to define the interaction among classes or properties.

Equivalent classes may be defined. It implies that both classes must contain the same set of individuals. Disjoint class can imply that a member of one class cannot be a member of the other class at the same time. Then, the complex class may be given from connectives such as complement, intersection, and union. A class can be defined as an enumeration of individuals.

A property maps from a domain of individuals (instances of a class) to a range of individuals. The domain and range may be from the same class. The property characteristics may be defined later. For example, it may be an equivalent property where “engage” and “play” mean the same thing in sentences such as “A person plays sports.” or “A person engages in sports”.

Inverse property and functional property are taken into consideration. Functional property means the property has no more than one output value. For example, a spa shop has one unique ID. Similarly, it is an inverse functional property. Thus, we can infer that two spa shops cannot have the same ID. The other characteristics are symmetric property. Transitive property creates the inferences between two properties. For instance, Chiva-Som is in Hua Hin and Hua in is in Prachuap Kirikun province. Thus, Chiva-Som is in Prachuap Kirikun province.

The property restrictions are also defined. The restrictions specify the condition of instances of the class. For example, it is the universal quantification (forall), existential quantification (for some), or specific values as well as cardinality restriction, where we can define max, min, and the number of exact individuals (in Table 1) (Cardoso 2006). The design guideline adopted is described in “Appendix 2”.

**Table 1:** List of ontology studied

Ontology name	Owner	*DL* Expressivity
travel.owl (Knublauch)	HolgerKnublauch	SOIN^(D)^
Etp-tourism (Mili et al. 2011)	PetkoValtchev	SHOIN^(D)^
e-tourism.owl (Siorpaes et al. 2004)	DERI (STI Innsbruck 2009)	ALCHIF^(D)^
Qall-me (Ou 2008)	STREP project (Oct 2006–Sep 2009) by FBK [Trento, Italy]	SHOIN^(D)^
Travel guides (Cunningham et al. 2014)	Travel Guides ontology	SHIF^(D)^

Protégé 4.3 is used for the ontology design and Hermit 1.3.7 and Pellet 2.3.1 reasoners are applied to check reasoning as we will describe in the next section.

**Fig. 2:**
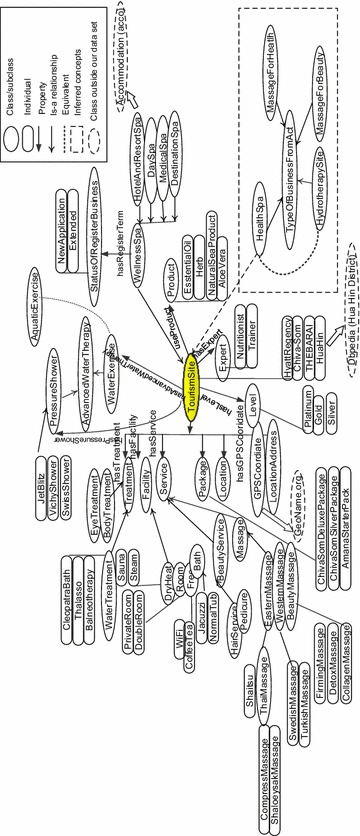
Main concepts

Figure 2 presents the overview of the ontology. It contains only important concepts. All classes are displayed in “Appendix 3”. Our main concept “TourismSite” is as highlighted in the center circle. Full documents are available at http://health-tourism.cpe.ku.ac.th/huahinonto/index.html.

In Fig. 2, the solid circles represent class and subclasses while the dashed circles show external concepts. Solid-headed arrows show property relation and lighter-head arrows show an *is*-*a* relationship (subclasses). The dashed line shows an example of equivalent classes.

Individuals of “TourismSite” are spa shops, Hua Hin, hotel name, and etc. Wellness spa is one class of *TourismSite*. WellnessSpa is divided into 4 subclasses: hotel and resort spa, day spa, medical spa, and destination spa. The spa shop may also be registered to the Ministry of Public Health. The registration can be a new application for the first year and extended status for the following year. Various kinds of concepts related to spas are shown such as services, facility, treatment, packages, and location. There are subclasses of services such as massage, and beauty service, and subclasses of facilities such as free services, room, and dry heat facility. For example, a massage can be divided into many types: eastern massage, and western massage where Thai massage is in the category of eastern massage. Beauty massage is such as slimming massage, firming massage, and collagen massage.

External concepts can also be linked such as linking GPS coordinate to *geonames* (http://www.geonames.org/ontology/documentation.html), Hua Hin individual to Hua Hin District in *dbpedia* (http://dbpedia.org/page/Hua_Hin_District), or HotelandResortSpa to an accommodation concept (http://ontologies.sti-innsbruck.at/acco/ns.html).

Consider the dashed box. This part presents the class of spa businesses based on the Act as in “Background” section which has three types: health spa, massage for health, and massage for beauty. Health spa is the focus where we will infer to equivalent class as *HydrotherapySite*. We can put axioms to define the valid health spa by considering individual services and facilities. Then valid health spas inferred will be a member of *HydrotherapySite.* Similarly, we can do the same thing to define a valid massage for beauty business or massage for health business. Note that one business can belong to more than one kind.

The above ontology is first evaluated by reasoners, namely Pellet and RacerPro, to check the correctness of the inferred results from defined axioms, and the usefulness of characteristics. Table 2 presents the measurement from various aspects. Metric row shows the summary of number of axioms, classes, properties and individuals. The DL expressivity is SRIOQ (Dentler et al. 2011).

**Table 2:** Ontology metrics

	DL expressivity	SRIOQ^(D)^
Overall metrics	Axiom	1799
	Logical axiom count	935
	Class count	80
	Object property count	92
	Data property count	16
	Individual count	250
Class axioms	SubClassOf axioms count	55
	EquivalentClasses axioms count	19
	DisjointClasses axioms count	3
	CGI count	0
	Hidden CGI count	19
Object property axioms	SubObjectPropertyOf axioms count	37
	EquivalentObjectProperties axioms count	3
	InverseObjectProperties axioms count	33
	DisjointObjectProperties axioms count	2
	FunctionalObjectProperty axioms count	10
	InverseFunctionalObjectProperty axioms count	1
	TransitiveFunctionalObjectProperty axioms count	2
	SymmestricObjectProperty axioms count	1
	AsymmestricObjectProperty axioms count	2
	ReflextiveObjectProperty axioms count	1
	IrreflextiveObjectProperty axioms count	3
	ObjectPropertyDomain axioms count	82
	ObjectPropertyRange axioms count	82
	SubPropertyChainOf axioms count	2
Data property axioms	SubDataPropertyOf axioms count	7
	EquivalentDataProperties axioms count	1
	DisjointDataProperties axioms count	1
	FunctionalDataProperty axioms count	3
	DataPropertyDomain axioms count	14
	DataPropertyRange axioms count	12
Individual axioms	ClassAssertion axioms count	274
	ObjectPropertyAssertion axioms count	196
	DataPropertyAssertion axioms count	64
	NegativeObjectPropertyAssertion axioms count	1
	NegativeDataPropertyAssertion axioms count	0
	SameIndividual axioms count	24
	DifferentIndividuals axioms count	0
Annotation axioms	AnnotationAssertion axioms count	417
	AnnotationPropertyDomain axioms count	4
	AnnotationPropertyRangeOf axioms count	4

We ran Pellet, and RacerPro to verify it which took 1671, and 17,630 ms respectively. The results contain approximately 32, 354 unsatisfiability class inference for Pellet and RacerPro respectively and there are 1, and 84 unsatisfiability object property inferences.

The domain concept is also verified by the domain experts who evaluated based on the completeness and accuracy of the terms. At last, the structure of ontology is commented by the ontology experts.

The questionnaires for the ontology experts are based on the above internal layers and external dimensions in the following section as well as the ontology dimension. We build ontology documents for experts to read. The documents are categorized in two kinds. First, it is the concept of the ontology which we hand to the domain knowledge expert to evaluate the completeness, and correctness of the terminology. Secondly, we construct the technical ontology document for the ontology expert to verify the architecture and structure of the ontology. The technical document is in the HTML form located at the site http://health-tourism.cpe.ku.ac.th/huahinonto/index.html.

For the domain experts, the following aspects are demanded: the completeness of each class, and the correctness in terms of naming, category, and description. The score is given in five levels [1, …, 5], where value 5 is the most proper value. The domain experts agreed to our domain concepts. The average score of the main classes from the two experts is 4.6. The overall correctness of the terminology and description is 4.59.

**Fig. 3:**
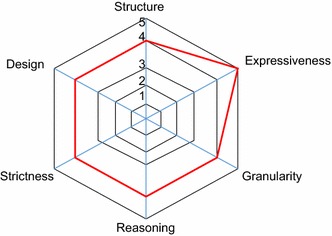
Ontology dimension score

From the ontology experts, the comments are all satisfactory. One expert agreed that the comment can be used via “*rdfs:comment*” but there are other choices of putting annotation such as using multi-language using “*rdfs:lang*”. To improve the usability, the comments can be translated in many languages. The depth of classes is about 2–3 levels from roots with *is*-*a* hierarchy. One good comment is the difference between “equivalent class” and “synonym”. In some cases, only a synonym is sufficient, such as “Sauna” and “Dry Heat”. It is better to put as two labels rather than two classes. Also, the similar problem is with “*sameAs*” individual. Attention should be paid to distinguishing between individuals and classes. There should not be lots of equivalent classes; the use of labels may be better. Besides, the object properties/characteristics are comprehensive. The ontology is a good starting point for the standard depending on its usability in the future. Figure 3 shows the ontology dimension score.

The ontology data set is linked to Link Open Data Cloud (Cyganiak and Jentzsch 2011; HPI 2011). We need to adjust several points for this application such as all the URIs in the data set must be resolved. Linked Open Data Cloud must be in RDF data format such as (RDFa, RDF/XML, Turtle, N-Triples). The data set should contain at least 1000 triples and must be connected via RDF links that are already in the diagram. The data set may be accessed using SPARQL endpoint, RDF crawling, or RDF dump. After the data set meets these criteria, it is added to the Data Hub located at http://datahub.io/organization/https-www-facebook-com-healthtourismmanagement as shown in “Appendix 4”.

## Hua-Hin health tourism application

The web application that exploits the health tourism ontology is developed. For the user interface, we gather requirements from stakeholders: administrative personnel who maintain spa shops’ web site, spa business managers who provide us their spa services, menus, and tourists.

**Fig. 4:**
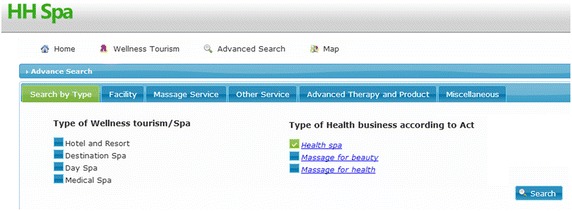
Example of user interface

The example of a web site is in Fig. 4 where a user is asked to search for Health Spa.

Let us consider the case of knowledge inference. According to the Act of Public Health Ministry mentioned in “Background” section, for the main concept, TourismSite, there are three types according to the Act: HealthSpa, MassageForBeauty, and MassageForHealth.

A health spa must have the use of water treatment, or advanced water therapy. In particular, the water treatment can be pool, tub, jacuzzi relaxation, bath, or foot bath. The advanced water therapy can be such as water exercise or aquatic exercise whose individuals can be Watsu, Aichi, and water relaxation. A health spa must contain at least one bath service and one pressure shower whose individuals are such as Effusion shower, Swiss shower, Vichy shower, Jet Blitz, and Experience shower.

The following axiom (1) describes the concept of health spa. Note that the property “*hasRegisteredTerm*” implies that the spa shop must be registered to the Ministry of Public Health. The registration can be the new registration or extended application for subsequent years.

1 $$\begin{aligned} & \text{`}\text{`}{\text{TourismSite}} \\ & {\text{and}}\;(({\text{hasAdvancedWaterTherapy}}\,\hbox{min} \,1\,{\text{AdvancedWaterTherapy}}) \\ & {\text{or}}\;({\text{hasWaterTreatment}}\,\hbox{min} \,1\,{\text{WaterTreatment}})) \\ & {\text{and}}\;({\text{hasBathType}}\;\hbox{min} \,1\;{\text{Bath}}) \\ & {\text{and}}\;({\text{hasPressureShower}}\;\hbox{min} \,1\;{\text{PressureShower}}) \\ & {\text{and}}\;\left({\text{hasRegisteredTerm}}\,\hbox{min} \,1\,\left(\left\{ {{\text{Extended}},{\text{NewApplication}}} \right\}\right)\right) \text{''}\\ \end{aligned}$$

**Fig. 5:**
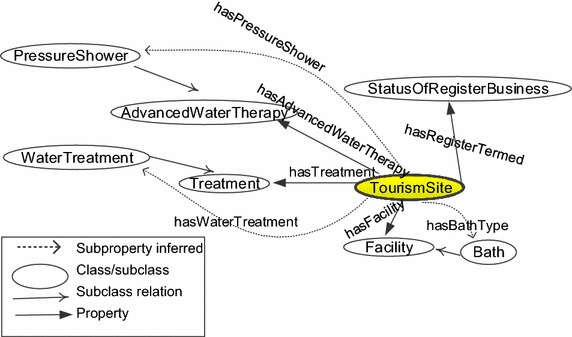
Related classes and properties for axiom (1)

Figure 5 shows the related classes, subclasses, and properties used by axiom (1). The dashed arrows are subproperties from properties. For example, *hasBathType* is a subproperty of *hasFacility* since *Bath* is a subclass of *Facility*.

**Fig. 6:**
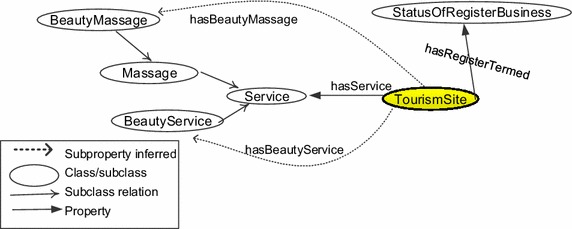
Related classes and properties for axiom (2)

For a beauty massage shop, the spa’s purpose is to massage for beauty improvement. The shop must provide beauty massage service including individuals such as firming massage, detox massage, fat massage, and collagen massage and the business shop has beauty services which are classified as nail services, hair services, and eye services. The equivalent class axiom is defined as axiom (2). Figure 6 displays the classes, subclasses, and properties for this axiom.

2 $$\begin{aligned} & \text{`}\text{`}{\text{TourismSite}} \\ & {\text{and}}\;({\text{hasBeautyMassage}}\;\hbox{min} \,1\;{\text{BeautyMassage}}) \\ & {\text{and}}\;({\text{hasBeautyService}}\;\hbox{min} \,1\;{\text{BeautyService}}) \\ & {\text{and}}\;\left({\text{hasRegisteredTerm}}\;\hbox{min} \,1\;\left(\left\{ {{\text{Extended}},{\text{NewApplication}}} \right\}\right)\right) \text{''}\\ \end{aligned}$$

We define *HydrotherapySite* which is equivalent class to *HealthSpa* as in the dashed box in Fig. 2, which can be inferred by axiom (1) and, thus, it is a subclass of *TypeOfBusinessFromAct*. Therefore, *HydrotherapySite* is also a subclass of *WellnessSpa*. The inferred individuals can be derived accordingly.

**Fig. 7:**
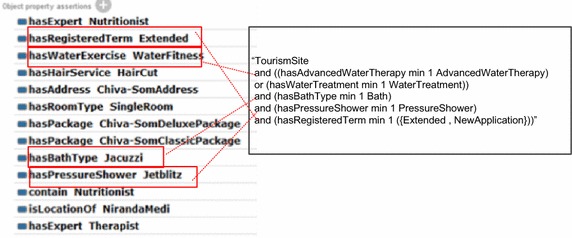
Chiva-Som properties and axiom evaluation

Consider Chiva-Som as an individual. Chiva-Som is the kind of desitnation spas in Hua Hin. It is registered as extended application, and property assertion and data properties are shown in Fig. 7. Thus, evaluating axiom (1) can result in “Chiva-Som is a HydrotherapySite”.

**Fig. 8:**
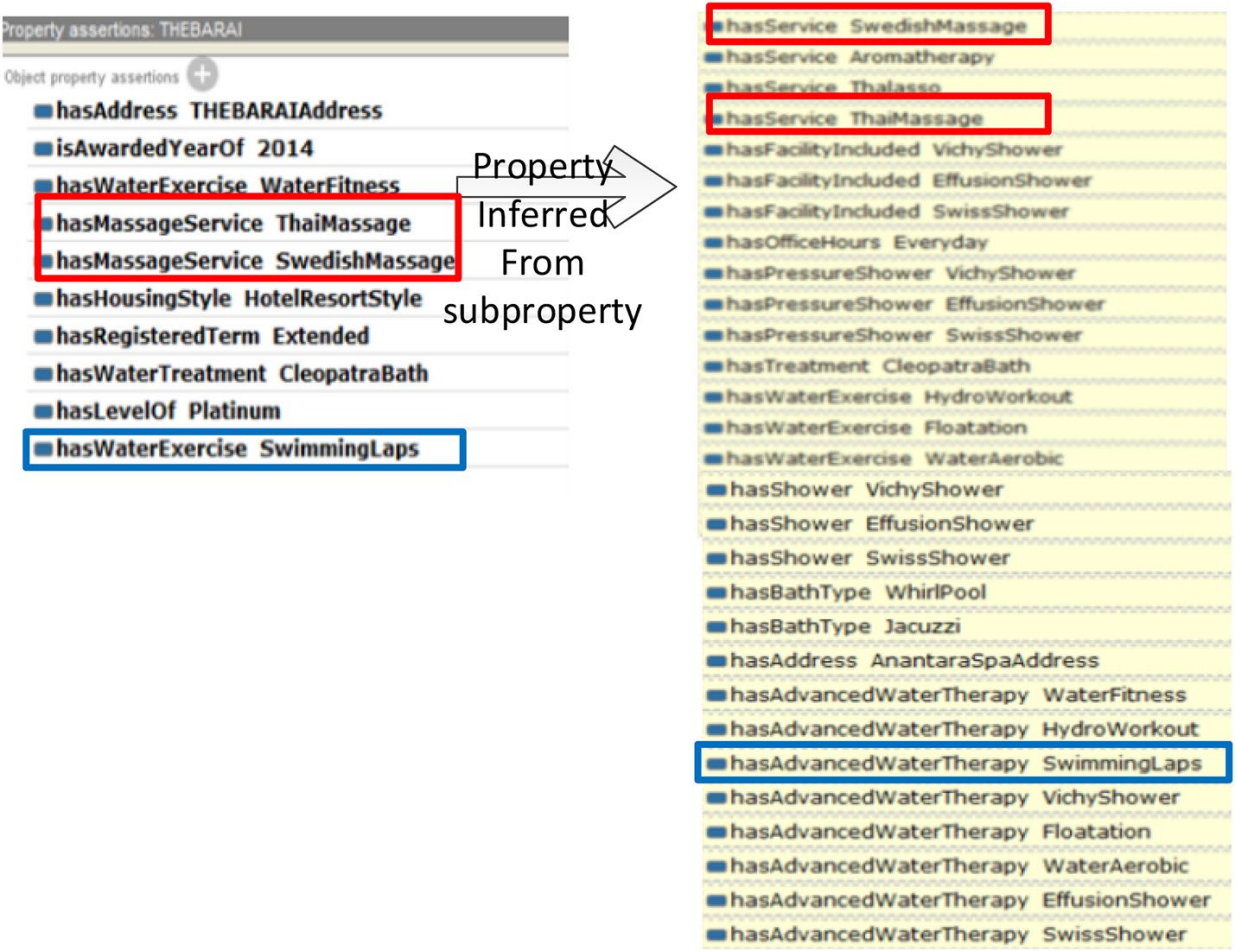
THE BARAI property inferred

**Fig. 9:**
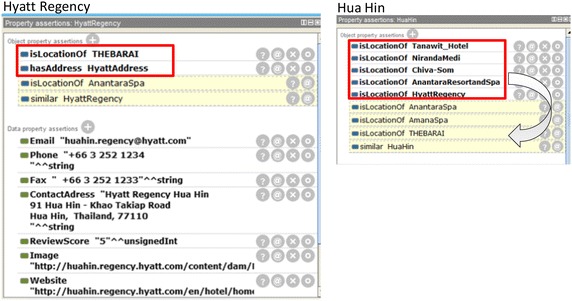
*isLocationOf* and *similar* property

In another example, THE BARAI individual and property assertions are similarly declared in Fig. 8. THE BARAI is a kind of hotel and resort spa. Figure 9 shows the example where properties are inferred from subproperties.

Figure 9 shows an individual, the Hyatt Regency Resort and Spa. To demonstrate the transitivity, consider the *isLocationOf* property. THE BARAI is a TourismSite located at the Hyatt Regency Resort and Spa in Hua Hin. AnantaraSpa is another spa in Hua Hin and Hyatt Regency is Hua Hin; therefore, is the location of Hua Hin, where *isLocationOf* has transitivity. The implication is “THE BARAI is located in this hotel, therefore, the address of THE BARAI is inferred from “LocationAddress” of this hotel by the object property “isLocationOf”. Similarly on the right side, Hua Hin is the location of Chiva-Som, Anatara Resort and Spa, Hyatt Regency, and etc. Consequently, Hua Hin is the location of THE BARAI, Anantara Spa, and etc.

*Similar* property has reflexivity; thus, we derive HyattRegency is similar to HyattRegency and Hua Hin is similar to Hua Hin.

## Lesson learned and discussion

Although the way we gather the information and construct the ontology is conventional, we believe that building semantic web application in each domain knowledge has specific characteristics and challenges. We have gained many interesting experiences regarding to this ontology construction, and the application.

- The study of domain knowledge is important. Without the correct knowledge, the information cannot be classified correctly in the first place.
  With the information collected, we learned that the health tourism concept in Thailand is very vague. Most people do not know the right meaning of spa. Medical tourism and health tourism are interchangeably used in many occasions.
  Most business shops are registered with the Ministry of Commerce. Only a few in the area are registered with the Ministry of Public Health as the business for Health (health spa, massage for beauty, and massages for health). The business may use the word “spa” in the title but it is not one of the three kinds.
- The ontology designed should be optimal. Individuals should not just be inserted in there and classified as subclasses. Object properties and characteristics are important to expand the knowledge by inference. The ontology should contain axioms, and equivalent classes to exhibit the rules for generating knowledge.
- The ontology design must have a goal. The axioms for classes can be used properly. For example, we may use the axiom to validate whether or not a business shop is a valid hydrotherapy site from their given services.
- Information gathering is a very important phase. If we cannot collect information properly, thoroughly, we cannot design the ontology to cover all of them.
- There is always room for improvement. Once we have more new data, the ontology may need to be revised. We can always find the wrong axiom or wrong inference results, due to wrong object properties/characteristics.
- Data cleansing is a huge task and it is never-ending. The naming is a major problem in every area. Even though with the standard dictionary, a business shop itself may use wrong words due to insufficient knowledge. Also, the local language is another problem. Thus, it is the problem of different ways of calling, spelling, and language translation.
- Besides, there are always new websites, web portals and new updates. The information gathering and maintenance is, therefore, the continuing process.

Comparison of our ontology to other tourism ontologies in Table 3 is presented in Table 2. Of all, *tgproton*, or PROTON (PROTo ONtology) contains the highest number of individuals. It covers upper-level ontology providing coverage of general concepts necessary for a wide range of tasks. The traveling domain can use http://goodoldai.org.yu/ns/tgproton.owl# prefix to gather the resources as proton.owl, travel_wkb.owl, and upproton.owl. The structure of these classes in owl file is about the traveler, attraction, destination, and user profile. Our ontology characteristics are shown in column “HT”. We start our design from the first version by adding more properties characteristics and axiom as well as individuals. The current version is evolved from *travel.owl* which is also a general tourism concept. Our HT can be integrated with existing general tourism ontology such as *dbpedia* (*Hua Hin*) i.e.:

- http://tourinthailand.org/ontology/healthtour.owl#Ahuahin => http://dbpedia.org/page/Hua_Hin_District

or the latitude or longitude can be mapped to *geoname*, i.e.,

- http://tourinthailand.org/ontology/healthtour.owl#GPSCoordination => geoname.org

Table 4 summarizes the reuse of standard prefixes: foaf, owl, rdf, rdfs, xsd in our ontology.

**Table 3:** Metric comparison of our ontology and other tourism ontologies

Metrics	E-tourism	Tgproton	Travel	ETP-tourism	HT
DL expressivity	ALCHIF^(D)^	SHIF^(D)^	SHOIN^(D)^	SHOIN^(D)^	SROIQ^(D)^
Total axiom counts	1535	12,306	145	907	1799
Total logical	1320	8135	93	533	935
Total classes	20	393	35	194	80
Total object properties	12	116	6	41	92
Total data properties	78	44	4	46	16
Total individuals	172	2855	14	19	250

**Table 4:** Ontology reuse prefixes

Value	Prefix	HT
http://xmlns.com/foaf/0.1/	foaf	–
http://www.w3.org/2002/07/owl#	owl	6812
http://www.w3.org/1999/02/22-rdf-syntax-ns#	rdf	4898
http://www.w3.org/2000/01/rdf-schema#	rdfs	4875
http://www.w3.org/2001/XMLSchema#	xsd	31,656

Table 5 shows the features’ comparison between the semantic web developed in this paper in the last column and existing systems of tourism semantic web. Our system provides a semantic web search engine concerning Hua Hin district and spa tourism domain. We used web crawler to gather information from various Hua Hin web portals. However, our system does not serve a commercial purpose like SATINE, and has not provided web services yet (which is an ongoing work), and Augmented Reality (AR) library has not support.

**Table 5:** The features comparison between the presented system in this paper and existing systems of tourism semantic web

Features	EIFFEL	Harmonize	SATINE	Bottari	Jakkilinki, Sigala et al.	Cardoso (2006)	Our work
Semantic search engine	–	–	–				–
Semantic web	–	–	–	–	–	–	–
Find relationship among the website							
Indexing							
Sorting							–
Ontology base		–					–
Business			–				
Web services			–			–	
Crawling from social networks–website to ontology				–			–
Augmented reality (AR)				–			
Tour planner				–	–		

## Conclusions

We present the ontology construction experience of Hua Hin Health Tourism, Thailand. The methodology follows the standard approach which starts from gathering Hua Hin heath tourism information. Two gathering approaches are used. The first one is an automatic information extraction from HTML documents which focus on the agencies like Agoda, TripAdvisor, HeyHuaHin and AtSiam. These sites contain mostly hotel information with spa facilities. The information from website is usually not sufficient. For other kinds of shops such as day spa shops, hospital, and clinics without official websites or not hosted at a large agency like Agoda or TripAdvisor, a field trip is also necessary. These shops may have only Facebook or FourSquare pages. Data from both gathering approaches are cleaned up, unified in many aspects, and merged automatically.

The study of corpus of health tourism is also regarded as a specification phase. Keywords, categories, and local laws related to it are investigated. The existing tourism ontology design and construction are also studied. Protégé’ is used as a tool to construct the ontology concept. Its plugin inference engine is used to check for the reasoning correctness. We also evaluate the ontology using the domain expert and ontology expert based on existing metrics both internal and external metrics.

At last, the application of the ontology is built. We are developing the semantic web on Health tourism for Hua Hin district. The example use of axioms is also shown for inferencing new knowledge.

## Figures and Tables

**Fig. 10:**
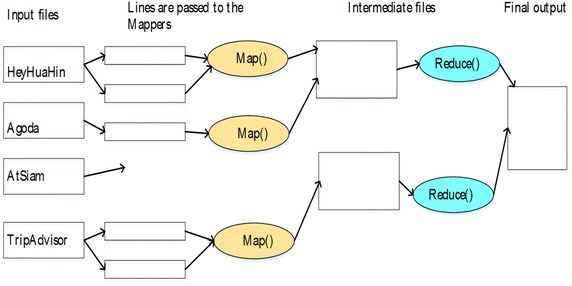
MapReduce phase

**Fig. 11:**
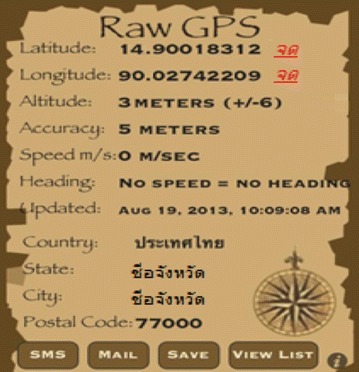
Latitude and longtitude recording from the smart phone with RawGPS. https://itunes.apple.com/th/app/raw-gps/id556374718?mt=8

**Fig. 12:**
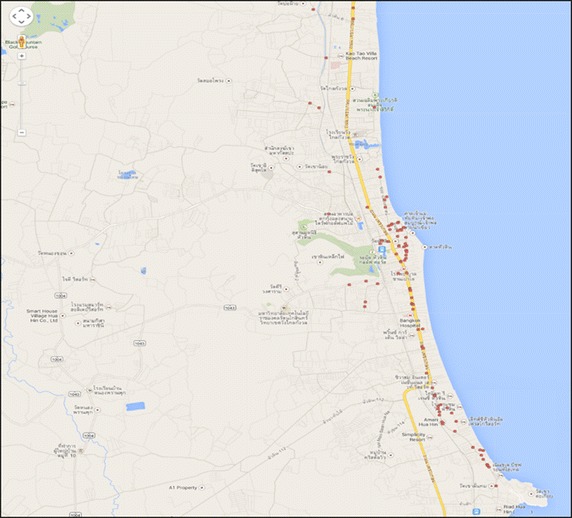
The field trip survey points for Hua Hin district for day spas

**Fig. 13:**
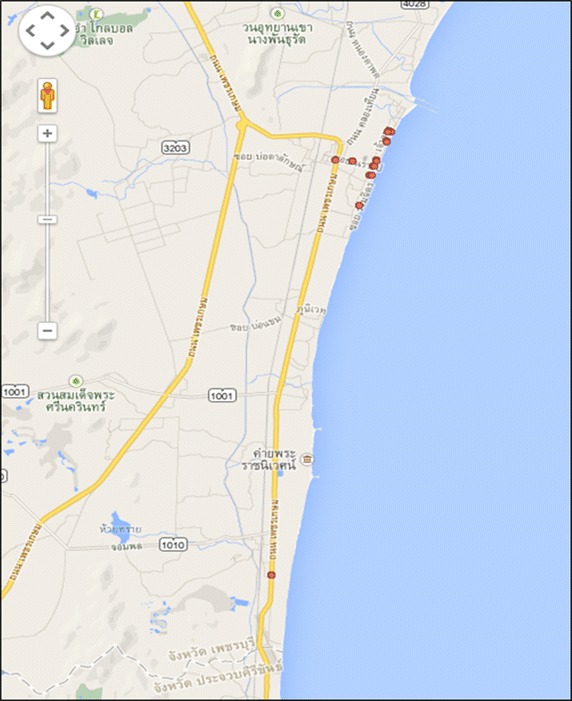
The field trip survey point for Cha-am district for day spas

**Fig. 14:**
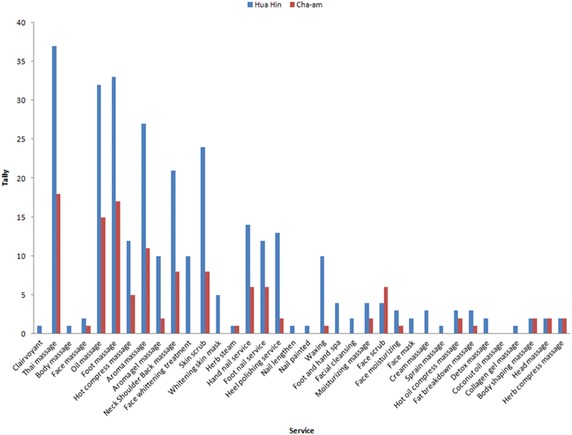
Categories of data collected

**Fig. 15:**
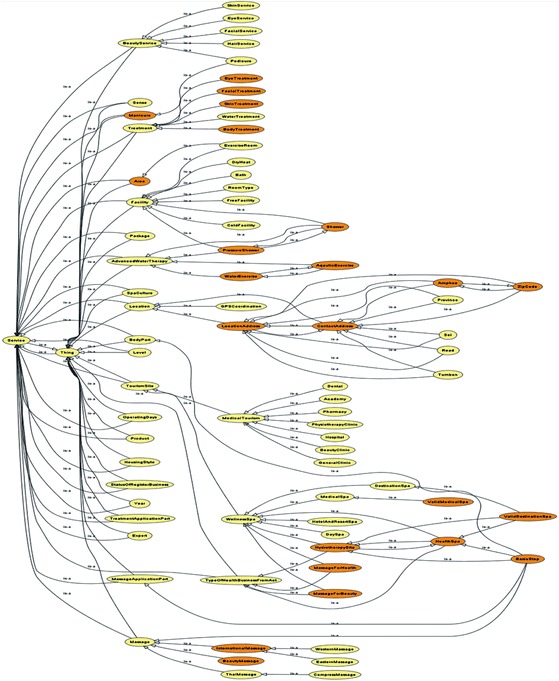
Overall concepts of the derived ontology

**Fig. 16:**
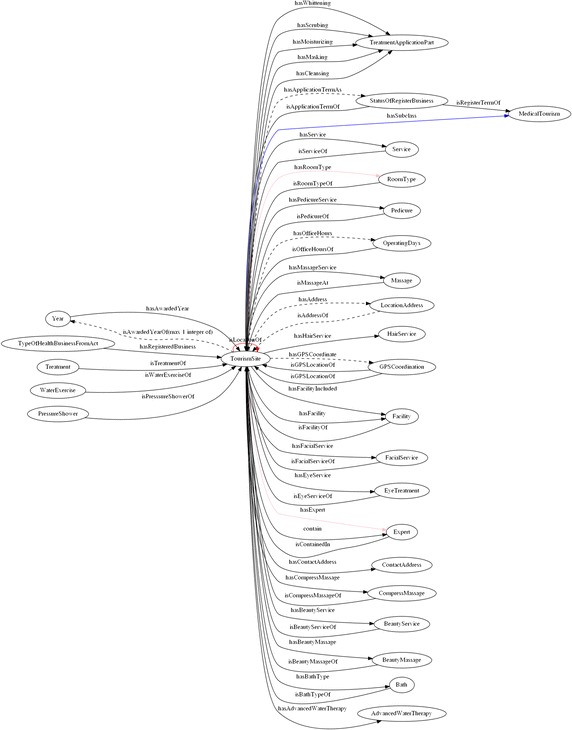
TourismSite concept

**Fig. 17:**
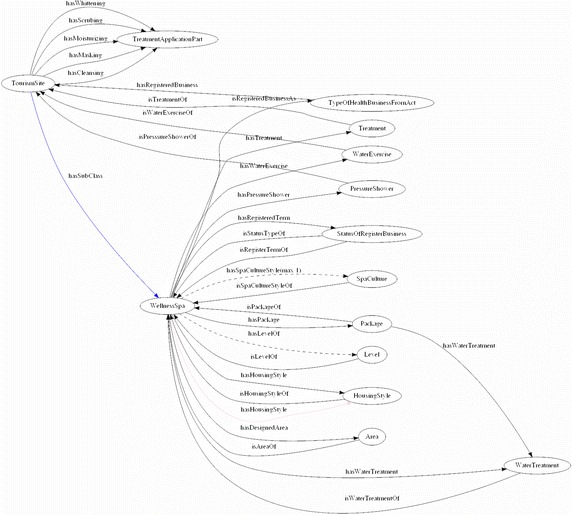
WellnessSpa concept

**Fig. 18:**
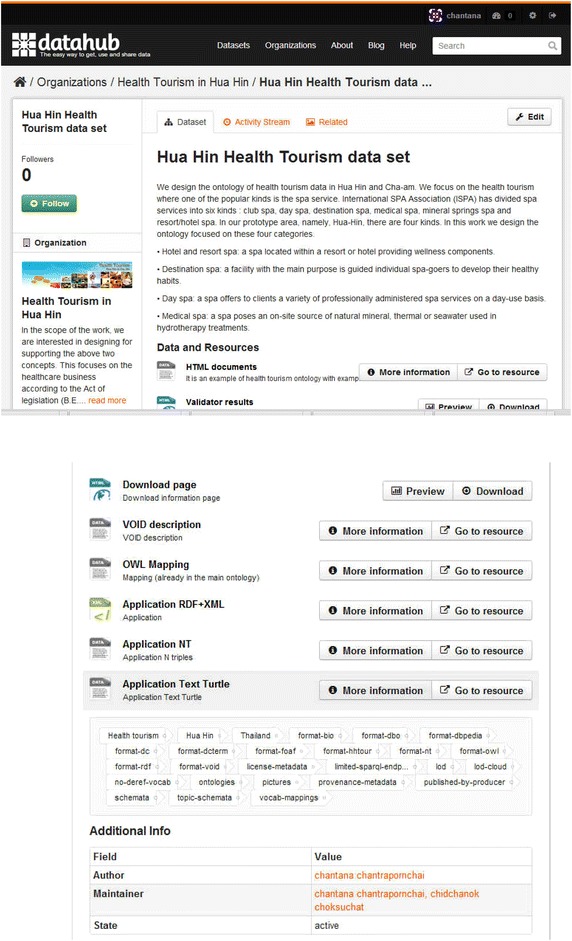
Health tourism ontology Data Hub at http://datahub.io/organization/https-www-facebook-com-health tourism management

## Appendix 1: Information gathering

There are two main sources of the data. The first one is from websites and social networks. The second one is the field trips and the collected hardcopy brochure.

### Web resources collector

As a case study, we have to collect the health tourism data of Hua Hin from several websites. The following popular tourism web site including Agoda (www.agoda.com), TripAdvisor (www.tripadvisor.com), AtSiam (www.atsiam.com), HeyHuaHin (www.heyhuahin.com) are explored. In particular, the hotel information from these websites that contain the spa facility are focused. For example, at Agoda website, it provides the spa as a facility of a hotel. For TripAdvisor, besides the hotel information, the day spa business can be found. It provides also the rating score of the spa within a given context of Hua Hin. For AtSiam, and HeyHuaHin, they are local web portals providing a list of hotel information in the area. Besides, we collect other information from social network like Facebook and FourSquare.

The information about the hotels and the spa facilities are collected as a text file from each website so they all will be ready to be merged. Each website corresponds to one text file with various following hotel attributes. The attributes may be the same or may not be the same for all the websites. All the text files have the hotel name in the first field.

Particularly, the hotel name is a key in the merging using MapReduce program. The values are the following information in the same line. The separators/markers are set for each field extracted. Then, we know which information is missing in the given record and which can be merged with the corresponding records from the other websites from the files.

We develop the program spawning the threads to crawl to each specified website to gather the information. The program is developed using Java Concurrency package in JDK 1.7. The thread writes the information found to the text file. All files are used to the next MapReduce phase after the data cleaning.

Using MapReduce can cope with large unstructured data in the key value form, such as the information extracted from the websites. Compared to using the regular approaches, the data has to be searched sequentially or imported into the database for quickly search. Sequential search for large text file is not practical while using database requires the fixed field formatting. Using key-value format is convenient and can be applied with the MapReduce paradigm which provides concurrent handling of large text files.

In Fig. 10 from left to right, the text file obtained from the Java concurrency program is read from disk and the mapper task, created my are created based on the input files. The default block size and one reducer task are used. The files are split to each mapper task. The reducer tasks merge the intermediate output from the mapper tasks.

Details of this experiments and the performance can be found at (Choksuchat et al. 2014). After the MapReduce phase, the data are formatted for the ontology extraction afterwards.

### Other resources

We have the students do the field trip gathering the information about health tourism business in the area since the data from the web portal is not sufficient and do not cover all kinds of the health tourism attractions. We also manually search spa packages and spa information from their official websites.

For the field trip, most of the case, we collect the information for day spas that are not located in hotels or resorts. Most of them are small local business. Also, shops in the shopping malls are needed to survey. Some shops are not registered with the Ministry of Public Health as the health spa. Rather, they are registered as the massage for health business or massage for beauty business. Some are only registered at the Ministry of Commerce.

The data for medical tourism such as beauty clinics, pharmacy, dental clinics, physiotherapy clinics, health care clinics, etc. are also collected. The field trip faces some difficulties. For example, some of the business owner did not wish to provide the students their information since they did not trust strangers. The students needed to walk around the area, collected the brochure, recorded posted prices, took pictures and recorded the GPS positions. The program RawGPS was used for recording GPS locations. Latitudes and longitudes of the shops were recorded as in Fig. 11.

Figure 12 shows the locations of the day spas collected and Fig. 13 are the locations of the day spas from Cha-am district. The medical care shop statistics are collected as shown in Table 6. The field trips are needed to perform again for double checking the data.

**Table 6:** Summary of data collected from field trips

Type	Hua-Hin district	Cha-am district
Spa	100	20
Pharmacy	48	4
Dental clinic	15	2
Blood laboratory	3	2
Physiotherapy	3	1
General clinic	20	1
Beauty clinic	2	–
Hospital	3	–
Healthcare academy	2	–

Figure 14 shows the services provided by the business. The service names were classified and groups to be in the concepts in the ontology. We recorded these data in an Excel file. The postal mails were sent to the business to request more information about their spa business. The request form was designed in both paper and Google form. The forms were sent to the all listed business.

### Data cleansing, unambiguity, name entity, and combining

The information about the hotels as well as their spa facility at various websites may not be consistent. For example, the names of the hotels should be consistent between various websites. This is important because it is used as a key in merging using MapReduce.

The same hotel may be named slightly different in various websites. For example, the name “Asara Villa & Suite Hotel Hua Hin” is used by Agoda, while TripAdvisor may use “Asara Villa & Suite (Hua Hin, Thailand)”. Another example is that Agoda may use “Anantara Hua Hin Resort & Spa” while Tripadvsior uses “Anantara Resort and Spa Hua Hin”. Besides the naming problem in the first field, other fields also have similar problems. Manual data cleaning is required. For the hotel names, we rely on the official hotel websites.

The names used are sometimes in English while some of them have Thai official web sites. This causes difficulty in finding information about the particular shop name. The manual searches are required to find the names in both English and Thai. Another example is the phone number field. Some has formatted with the country and area codes while some has formatted with only the area code, etc. For searching the information on the social network, such as the number of likes in their Facebook pages, the rating in the FourSquares, or the ranking in TripAdvisors, the way to obtain them are very dependent to their web services.

For the spa service name or treatment name, the synonym is a usual problem. For example, Aromatherapy is a kind of massage therapy that uses natural compound product with aromatic sense for relaxing minds, mood and body. Some business may call it as Aroma massage, referred to the Aromatherapy. Another example is Japanese bath. It is also called Shaistu. For sauna facility, it is a dry heat facility. Some countries call it, Laconicum (i.e. Spartan). We have to call the facility in many ways according to the dictionary and definitions found. This needs an in-depth study of the terminology in the area. We studied the literatures of the health tourism from many textbooks and many web resources especially for Thai massage or for Thai spa. The keywords and terms are collected, grouped and categorized.

The text files storing these collected data in the CSV format are merged using Google Refine to save into the ontology format. After importing to the ontology using Google Refine, the property editing for classes are required.

## Appendix 2: Design guideline

The careful consideration for designing the ontology is summarized according to the following issues:

1. Classes or individuals or label: Without experiences, it is hard to decide which keywords are class names and which ones are individuals or just labels. The class names should be entities that contain instances. There should be classes underneath owl:Things, describing various concepts. The classes may have subclasses which are subcategories. Under classes/subclasses, one should be able to define individuals or instances under them. If it is a label, it is just another name or annotations.
2. Object property: It shows relationships between classes. The properties can also have subproperties which can be useful for inference from subclasses. The properties can be inversed. The multiplicity or restrictions on its domain and range can be setup. To decide the property restriction, as well as chaining property are not easy tasks.
3. Property characteristics: It describes as: reflexive, symmetric, transitive, irreflexive, functional, inverse function, etc. The characteristics help the inference engine well if defined properly. This enable inferring new knowledge. The difficulty is that the designer needs to understand the meaning of these characteristics, and the applicable domain and range so that the wrong results of the inferred axioms do not occur.
4. Data property: It is often confusing between data properties and classes. The data property is the attributes of the classes. It can be the primitive data type as well the structures of many fields of primitive data types. One needs to decide whether the given name is a data property or class or individual or a label of an item.
5. Assertions/equivalent classes: For a class, we can construct a new class based on the existing ones by using equivalent classes. Object property can define to be an equivalent class as well. For individuals, we can define assertions describing the facts about individuals.
6. Reasoning: It depends on the reasoners we use. The reasoner can check the validity and verify the property/axiom/classes/individual inferences. We have to check whether the inference results are correct. Without care, the cycle of axiom/assertion may occur. Also, specifying the wrong domains and ranges can lead to the wrong inference results. Some reasoner may be breakdown during the inference.

After all these, the good design will lead to the search that yields precise results. Moreover, new conclusion may be derived with the reasoning capability. This increases the power of semantic web query over the traditional web with more meaningful property characteristics, assertion, equivalent classes and axioms. It is usually questionable whether it is worth to build the ontology for certain application. This depends on the data set we are exploring. If the data set is complex, and can be grouped into many subclasses and have many relations, it may be worth to use to ontology design. The semantic query language like SPARQL will process the query and inferences. The programmer needs not to hand-code the derivation in the database or program. In the other words, using the ontology, we may spend time on the design longer but with the support reasoning engine, we can shorten the implementation as well as the data structure maintenance time.

## Appendix 3: Detailed classes

Figure 15 presents the big picture of ontology while Fig. 16 presents only TourismSite concept. Figure 17 is WellnessSpa concept. The full document is attached in Supplementary II.

## Appendix 4: Published ontology

Figure 18 is the published ontology on datahub.
